# 

**Published:** 1841-06

**Authors:** 


### WESTERN JOURNAL

OF

### MEDICINE AND SURGERY.

### COLLABORATORS.

Edward H. Barton, M. D. *Professor of the Theory and Practice of Medicine, in the Medical College of Louisiana.*

William J. Barbee, M. D. *Cincinnati, Ohio.*

George W. Bayless, M. D. *Demonstrator of Anatomy in the Louisville Medical Institute.*

Solon Borland, M. D., *Memphis, Tennessee.*

A. H. Buchanan, M. D. *Columbia, Tennessee.*

Charles Caldwell, M. D. *Professor of the Institutes of Medicine and Medical Jurisprudence in the Louisville Medical Institute.*

Samuel A. Cartwright, M. D. *Mississippi.*

A. Clapp, M. D. *New Albany, Indiana.*

Jedediah Cobb, M. D. *Professor of Anatomy in the Louisville Medical Institute.*

John Esten Cooke, M. D. *Professor of the Theory and Practice of Medicine in the Louisville Medical Institute.*

Samuel D. Gross, M. D. *Professor of Surgery in the Louisville Medical Institute.*

John Hardin, M. D. *Munfordsville, Ky.*

John P. Harrison, M. D. *late Professor of Materia Medica, in the Cincinnati College.*

John F. Henry, M. D. *Bloomington Illinois*

Samuel P. Hildreth, M. D. *Marietta, Ohio.*

Samuel Hogg, M. D. *Nashville, Tennessee.*

M. Z. Kreider, M. D., *Lancaster, Ohio.*

Moses L. Linton, M. D. *Springfield, Kentucky.*

Henry Miller, M. D. *Professor of Obstetrics and the Diseases of Women and Children in the Louisville Medical Institute.*

John W. Monette, M. D. *Washington, Mississsippi.*

Samuel B. Richardson, M. D., *Lecturer on Anatomy and Surgery, &c, Louisville, Ky.*

John L. Riddell, M. D. *Professor of Chemistry in the Medical College of Louisiana.*

Landon C. Rives, M. D. *late Professor of Obstetrics in the Medical Department of the Cincinnati College.*

Charles W. Short, M. D. *Professor of Materia Medica in the Louisville Medical Institute.*

G. Troost, M. D. *Professor of Chemistry and Mineralogy in the University of Nashville.*

Amasa L. Trowbridge, M. D. *Professor of Surgery, Willoughby University, Ohio.*

John A. Warder, M. D. *Cincinnati, Ohio.*

William Wood, M. D. *Cincinnati, Ohio*

### INDEX TO VOL. III.

A

Abscess Hepatic, Dorris' Case of, - - - - 352
Abscess Suppurative, Utilility of Tonico-Astringent Injections in, - - - - 343
Almanac, American Medical for 1841, - - - - 75
Aneurism, Axillary, - - 320
Axillary Aneurism, Gross on, 401
Ani Prolapsus, New Operation for - - - - - 145
Arsenic, Poisoning by, 227
Arsenic, Poisoning by, treated with Hydrated Peroxide of Iron, - - - - 55

B

Bandages, Henkel on, - 379
Bell's Case of Phlebitis, 149
Bronchiæ Obliteration of, Reynaud on, - - 241–321
Buchanan's Case of Peritonitis, - - - - - 101
Buchanan,s Case of Triplets, 253

C

Caldwell, Professor, - 398
Card, - - - - - 397
Cheiloplastic Operation, 385
Colocynth Oil, A Substitute for Croton Oil in Neuralgia, Sciatica, &c. - - - - 63
Congenital Deafness, relieved by an Operation, - 95
Connecticut Retreat for the Insane, - - - - - 235
Cooper, Sir Astley, Death of, 320

D

Danish Medical Statistics, 64
Deafness Congenital, relieved by an Operation, - - 95
Diagnosis, Raciboski's Elements of, - - - 400
Drake on Milk Sickness, 161
Dublin Dissector - - 381
Dublin, Medicine in, - - 315
Death of Dr. Wm. P. Dewees. 479
Dorris' Case of Hepatic Abscess. - - - - - 352

E

Ergot, Oil of, External Application of, - - - 142

F

Fever, Autumnal, Halloway's Case of, - - - - 347
Fever, Bilious, Hardin's Observations on, - - 104
Fever, Bilious and Congestive, Keller on, - - 159
Fever, Remittent, pain of the back in, - - - 141

G

Gallup's Institutes of Medicine, - - - - 113
Geological Voyage, 399
Green, Case of Suppurative Abscess, - - - 343
Gross' Case of Pretended Mayhem, - - - - 355
Gross on Axillary Aneurism, 401
Gross, Professor, - - 239

H

Hardin on Bilious Fever, 104
Harrison, President, death of, 398
Harrison, President, Case of, 469
Henkel's Treatise on Bandages, - - - - - 379
Hepatic Abscess, Dorris' Case of, - - - - - 352
Hermaphrodite, Anatomical Description of, - - 386
Hernia, New Operation for the Radical Cure of, - - 384
Hernia, Strangulated, new facts in regard to, - 146
Holloway's Case of Autumnal Fever, - - - 347
Hooping Cough, Cold Affusions in, - - - 306
Hospitals of Paris, - 65
Humerus, Congenital Dislocation of, Reduced after Sixteen Years, - - 395

I

Insane, Connecticut, Retreat for, - - - - - 235
Institutes of Medicine, Gallap's, - - - 113
Iron, Lactate of, on the Employment of, - - - 62

K

Keller on Bilious and Congestive Fever, - - 159
Kidneys, Congestion of, 64

L

Landouzy on Varicocele, 357
Lee on some of the most important Diseases of Women, - - - - 271–453
Leonard's Case of Pregnancy, 87
Linton, on Medicine in Dublin, 315
Louisville Medical Institute, 80

M

Malgaigne's Operative Surgery, - - - - - 371
Mayhem, Pretended Case of, 355
M'Clintock's Introductory Lecture, - - - - 237
Medical Almanac American for 1841, - - - 75
Medical Convention of Kentucky, - - - - 157
Medical Convention of Ohio, 397
Medical Hoax, - - - 399
Medical and Physiological Commentaries, Paine's, 257
Medical Schools, - - 397
Medical Traveller, Desultory Notes, - - - 81
Medicine in Dublin, - 315
Medical Society of Tennessee. 478
Menstruation, Complete Absence of, - - - 63
Mercury, Solution of By-chloride in Itching, - - 143
Milk Sickness, Drake on, 161
Movements of Medical Men. 478

N

Nerve, Trigeminus, Irritation of a Branch of, - - 310

O

Obliteration of the Bronchiæ, Memoir on, by Reynaud, 321
Our Undertaking. - - 477

P

Paine's Medical and Physiological Commentaries, - 257
Paralysis Local, Treated with Phosphorus, - - 144
Peritonitis, Buchanan's Case of, - - - - - 101
Phlebitis, Bell's Case of, 149
President Harrison, Case of, 469
Phosphorus, in Treatment of Local Paralysis, - - 144
Poisoning by Arsenic, 227
Poisoning by Arsenic, Treated with Hydrated Peroxide of Iron, - - - - 55
Precocity in a Boy, - 61
Pregnancy, Leonard's Case of, 87
Prize Essay. - - - 480
Prolapsus Ani, New Operation for, - - - - 145

Q

Quinine, Sulphate of, in Enlargement of the Spleen, and Dropsies after Ague, 305

R

Rachitis, - - - 74
Rachitis, Memoir on General Characters of, - - 1
Raciboski's Elements of Diagnosis, - - - 400
Reynaud on Obliteration of the Bronchiæ, - - - 321
Rhinoplastic Operation, 385

S

Scrofulus Diseases, Causes of, 143
Stokes' and Bell's Practice, 79
Strabismus, Operation for, 240
Strabismus, Operations for, 59
Strangulated Hernia, New Facts in Relation to, 146
Surgery, Malgaigne's Operative, - - - - 371

T

Testicle, Diseases of Treated by Compression, - 147
Tetanus Traumatic, Wilson's Case of, - - - 91
Tetanus Traumatic, successfully treated by Tobacco Enemas, - - - 308
The Spring. - - - 478
Tonico-Astringent Injections, in Suppurative Abscess, 343
Torticollis, New Species of, 383
Trigeminus Nerve, Irritation of a Branch of, - - 310
Triplets, Buchanan's Case of, 253

V

Varix and Varicocele, New Method for the Radical Cure of, - - - - 393
Varicocele, Landouzy on, 357
Variola, Case of Posthumous, 314
Velpeau's New Method of Operating for the Radical Cure of Hernia, - - - 384

W

Wilson's Case of Traumatic Tetanus, - - - 91
Women, Lee on some of the most Important Diseases of, 271

### RECEIPTS FOR THE MEDICAL JOURNAL,

*For the month ending May 31st, 1841.*

| | |
|---|---|
| Dr. J. Bainbridge, Princeton, Ky. | $5 00 |
| " T. B. Johnson, Freedonia, Ky., | 5 00 |
| " J. A. Green, Covington, Tenn., | 5 00 |
| " G. W. Riddell, Knightstown, Ia. | 5 00 |
| " C. J. Randolph, Grove Hill, Ala., | 5 00 |
| " F. Taylor, Westport, Ky. | 5 00 |
| ' J. Westerfield, Glasgow, Ky., | 5 00 |
| " A. C. Lewis, Scott P. O., Ohio, | 5 00 |
| " Bierce, Athens, Ohio, | 5 00 |
| " R. S. McFarland, Hebardsville, Ky., | 5 00 |
| Drs. Porter & Whittun, Rockford, Ia , | 5 00 |
| Dr. W. H. Rudd, Perry, Ga , | 10 00 |
| " D. B. Rice, Macomb, Ill., | 5 00 |
| " W. P. Wallingford, Mason, Mo., | 5 00 |
| " S. Gregory, Stephenson, Ill | 5 00 |
| " Walter, Canton, Ill,, | 5 00 |
| " J. T. Wilson, Carr's P. O., Tenn., | 5 00 |
| " A. A. Burlesson, Decatur, Ala., | 5 00 |
| " H. H. Sugg, Keysburg, Ky., | 10 00 |
| " M. Johnson, Roscoe, Ohio, | 5 00 |
| Drs. Winans & Dawson, Jamestown, Ohio, | 5 00 |
| " Crary & Baxter, Columbia, Ia., | 4 50 |
| Dr. E. H. Davis, Chillicothe, Ohio, | 8 00 |
| " H. Brown, Davenport, Iowa, | 5 00 |
| " R. Nickle, Waidsboro, Ky., | 5 00 |
| " A. H. Buchanan, Columbia, Tenn., | 5 00 |
| " J. Kirkwood, N. Madrid, Mo., | 5 00 |
| " T. A. Webster, Campbellsville, Ky , | 5 00 |
| " G. R, Follis, Pleasureville, Ky. | 5 00 |
| " J. Pricketts, Centreville, Ia. | 5 00 |
| " J. Pennington, Milton, Ia., | 7 50 |
| " T. S. Davidson, Perryville, Ia., | 5 00 |
| " Dunlap, Indianapolis Ia., | 5 00 |
| " E. S. Williams, Cincinnati, Ohio, | 10 00 |
| " Strader, " " | 10 00 |
| " W. Richards, " " | 8 00 |
| " J. Martin, Xenia, Ohio, | 10 00 |
| " W. R. Winton, Hamilton, Ohio, | 8 00 |
| " L. Rigdon, " " | 10 00 |
| " Falconer, " " | 5 00 |
| " J. Fithian, Oxford, " | 5 00 |
| " J. T. Johnson, N. Richmond " | 5 00 |
| " J. Beasley, Ripley, " | 3 00 |
| " J. T. Moxley, Wheelersburg, Oho, | 8 00 |
| " M'Clelland, Richmond, Ky., | 10 00 |
| " J. F. Fleming, Elizaville, Ky., | 5 00 |
| Drs. Taliaferro & Marshall, Maysville, Ky., | 10 00 |
| Dr. G. E. French, Alexandria, La , | 12 50 |
| " H. S. Levert, Mobile, Ala., | 10 00 |
| " G. Venable, Hopkinsville, Ky., | 5 00 |
| " W. P. Jones, Bowling Green, Ky., | 5 00 |
| " F. D. Lewis, Hawesville, Ky., | 3 00 |
| " C. H. Preston, Paris, Mo., | 5 00 |
| " Richardson, Jefferson, Tenn., | 5 00 |
| " L. Lewis, Osceola, Mo , | 5 00 |
| " Quisenberry, N. Middletown, Ky. | 5 00 |
| " R. F. Brown, Spring Hill, Tenn., | 5 00 |
| " J. Thistle, Natchez, Miss., | 5 00 |
| " J. H. Dunlavy, Beattie's Bluff, Miss., | 5 00 |
| " D. G. Dedman, Lawrenceburg, Ky., | 10 00 |
| " M. Williams, Owensboro', Ky., | 5 00 |
| " R. Murray, Brushy Fork, Ky., | 5 00 |
| " A Crow, Hartford, Ky , | 5 00 |
| " Ashby, Ashland, Ky., | 5 00 |
| " Berry, Locustport, Ky. | 7 50 |
| " Swan, Henderson, Ky., | 5 00 |
| " W Fry, Middletown, Ky., | 5 00 |
| " J. Clayton, Christianburg, Ky., | 5 00 |

### TO DELINQUENTS.

Subscribers will observe that there has been quite an improvement in the length of the paying list. Seventeen hundred dollars still are lacking to refund our actual expenditures, and enable us to pay the editors. We trust that the pay list will be kept up to its present size, and that if we are not altogether saved the trouble of publishing a delinquent list, it may at least prove a short one.

☞ Remit by mail, at our risk, under the frank of the Postmaster; who, under the regulations of the P. O. Department, will in all cases frank letters containing subscription money.

☞ No subscription to commence but with the commencement of a volume. No subscription can be discontinued until the end of the year, and none, after one number of a given volume has been mailed, until the end of the volume.

PRENTICE & WEISSINGER.

---

The Western Journal of Medicine and Surgery is published monthly by the undersigned, at the corner of Main and Fifth streets, Louisville, at $5 per annum, payable in advance. Each number contains from 80 to 84 pages making two volumes in the year of about 500 pages.

Contributions to its pages by the Physicians of the Valley of the Mississippi, addressed to the Editors, are respectfully solicited.

Letters on business, to be addressed, postage paid, to the publishers.

☞ Postmasters, by the regulations of the Postoffice Department, will frank letters containing subscription money, and all remittances so franked are at the risk of the publishers.

*July* 25, 1840. PRENTICE & WEISSINGER

